# Metabolic syndrome in menopausal transition: Isfahan Healthy Heart Program, a population based study

**DOI:** 10.1186/1758-5996-2-59

**Published:** 2010-10-05

**Authors:** Ramin Heidari, Masoumeh Sadeghi, Mohammad Talaei, Katayoun Rabiei, Noushin Mohammadifard, Nizal Sarrafzadegan

**Affiliations:** 1 Department of Cardiology, Isfahan University of Medical Sciences, Isfahan, Iran; 2 Isfahan Cardiovascular Research Centre, Isfahan University of Medical Sciences, Isfahan, Iran; 3 Research Methodology and Biostatistics, COX Research Group, Poursina Hakim Research Institute, Isfahan, Iran; 4 Rehabilitation Department, Isfahan Cardiovascular Research Center, Isfahan University of Medical sciences, Iran; 5 Nutrition Department, Isfahan Cardiovascular Research Center, Isfahan University of Medical Sciences, Isfahan, Iran

## Abstract

**Introduction:**

There is a remarkable increase in cardiovascular disease after menopause. On the other hand, metabolic syndrome as a collection of risk factors has a known effect on cardiovascular diseases. Hormone changes are considered as one of the main relevant factor regarding cardiovascular disease as well as some recognized relationship with metabolic syndrome's components. This study was carried out in order to search for prevalence of metabolic syndrome during menopausal transition.

**Method:**

In a cross sectional study in urban and rural areas of Isfahan, Najafabad and Arak cities, 1596 women aged more than 45 years were investigated using Isfahan Healthy Heart Program's (IHHP) samples. Participants were categorized into three groups of pre-menopause, menopause and post-menopause. Leisure time physical activity and global dietary index were included as life style factors. The association of metabolic syndrome and its components with menopausal transition considering other factors such as age and life style was analyzed.

**Results:**

there were 303, 233 and 987 women in premenopausal, early menopausal and postmenopausal groups respectively. Metabolic syndrome was found in 136(44.9%) premenopausal participants and significantly increased to 135(57.9%) and 634(64.3%) in early menopausal and postmenopausal participants respectively, when age was considered (P = 0.010). Except for hypertension and hypertriglyceridemia, there was no significant difference between three groups of menopausal transition when metabolic syndrome's components were considered.

**Conclusion:**

In contrary to the claims regarding the role of waist circumference and blood glucose in increasing of metabolic syndrome during the menopausal transition, this study showed this phenomenon could be independence of them.

## Introduction

Today, cardiovascular disease is one of the main causes of mortality of women in the world [1]. Considering the metabolic changes that occur in women during post menopause (PM), there is an increase in risk factors of cardiovascular diseases (CVD) and incidence of these diseases in PM [2]. There are different points of view about incidence of CVD during PM period and their relationship. Some believe that the increased incidence occurs just by increased age but some studies such as Framingham demonstrated the fourfold increase in incidence of CVD in PM period [3]. Moreover, the incidence of CVD increases in patients with premature menopause or those who underwent surgeries that result in menopause [4]. Metabolic syndrome (MetS) is a cluster of components which make the individual susceptible to CVD. These components are: increased lipid accumulation in central parts of the body (abdominal obesity), insulin resistance (IR), dyslipidemia (elevated triglyceride and LDL and decreased HDL), and hypertension. According to NCEP-ATP III criteria (National Cholesterol Education Program- Adult Treatment Panel III), presence of at least three o three components in considered MetS [5]. Various studies have shown that being affected by MetS increases the risk of CVD as well as morbidity of the disease [6].

Increased incidence of MetS during PM period has been shown in many studies throughout the world [7,8]. A sixty percent increased risk of MetS and increased incidence of MetS after adjusting various background factors is observed in PM [9]. The etiology of MetS is not clearly defined, but it is shown that the syndrome is associated with visceral obesity [10]. Thus, the theory of metabolic changes during PM and increased abdominal obesity as a result of decrease in estrogen is one of the hypotheses which explain the increased incidence of the syndrome during this period [11]. Concerning the prevalence of CVD in women (about 22%) [12,13] and the prevalence of MetS (35-58%) in Iran [14,15], this study is aimed at determining the prevalence of the syndrome and its components in PM period and comparing it with that of the pre-menopause and menopause period.

## Method

The survey was conducted in 3 cities in the central part of Iran (Arak, Isfahan and Najafabad) as baseline survey of Isfahan Healthy Heart Program. The subjects were selected using multi-stage random sampling with clusters in the urban and rural areas. The project team conducted the baseline survey of 12,514 adults aged≥19 years. The number of individuals was in accordance with the total population of the 3 cities and clusters. Having resided for more than 10 years in these cities, not being pregnant for females and being mentally healthy were the criteria for participating in this study. Written informed consent was obtained from subjects after full explanation of the procedure involved. The study was approved by the ethics committee of the Isfahan University of Medical Sciences. The details of the program have previously reported elsewhere [16].

Initially, a questionnaire was completed at each subject's home by trained nurses.

It contained demographic information, smoking, menopausal status, consumption of relevant medication especially anti-diabetic agents, anti-hypertensive agents and hormone replacement therapy. In addition, food frequency questionnaire and a questionnaire for leisure time behavior were used for participants. Afterward, participants were invited to certain health centers, where blood samplings and clinical check-ups after 12 to 14 hour fasting were carried out. In addition to fasting blood glucose (FBG), 2-hour plasma glucose (2hPG) was also measured after 2 hours and after consumption of 75 g of glucose. Serum lipids, including total cholesterol (TC) and triglyceride (TG), were also measured using the relevant fasting blood sample. All the blood sampling procedures were performed in the central laboratory of the Isfahan Cardiovascular Research Center using the enzyme-linked method. Subjects were weighed with light clothes and no shoes, and their waist and hip measurement and weight were also noted. In addition, waist circumference (WC) was measured at a level midway between the lower rib margin and the iliac crest to the nearest half-centimeter. The blood pressures of the individuals were measured twice with a standard barometer, with the subjects in a sitting position, and the average blood pressure was taken into consideration.

The subjects of the present study were 1596 women, among all participants of the IHHP, who were classified into three groups: premenopausal women, aged 40 and over and still menstruate, early menopausal women with a permanent cessation of menses (spontaneous or surgical) of over 12 months and less than 3 years, and postmenopausal women who had at least a 3-year history of cessation of menses (spontaneous or surgical). The 44(2.8%) smokers and those currently used hormone replacement therapy 29(1.8%) were excluded the same as one other study did [26]. No participants used oral contraceptive pill or other hormone replacement therapy.

A modified definition by the Adult Treatment Panel III guideline of the National

Cholesterol Education Program16 was used to categorize the subjects according to the number of components of the metabolic syndrome. The metabolic syndrome was defined as the presence of 3 or more of the following components: 1) serum triglycerides ≥150 mg/dL; 2) HDL cholesterol ≤50 mg/dL; 3) glucose ≥100 mg/dL fasting or on treatment; 4) blood pressure ≥130/85 mmHg or anti hypertensive medication use, and 5) waist circumference ≥88 cm [10] The 2001 definition identified fasting plasma glucose of ≥110 mg/dL as elevated. This was modified in 2004 to be ≥100 mg/dL, in accordance with the American Diabetes Associations updated definition of impaired fasting glucose (IFG) [17]. Considering physical activity, the period and frequency of doing each specific activity as leisure in a week were asked. Leisure time physical activity was calculated in the unit of Mets minutes per day using extent coefficients of each particular activity [18] multiplying duration of performing it in a day. One MET is reflective of energy expenditure during rest [19]. Dietary behaviors were assessed with a validated, qualitative, forty-eight-item FFQ. The FFQ was adapted from the CINDI programme questionnaire [20]. A global dietary index (GDI), expressing global diet quality, was created by calculating the average of the mean of twenty-nine frequency questions in seven categories. Higher scores on the GDI represent diets higher in total fat, saturated fat and cholesterol. More details were depicted elsewhere [21].

### Statistical Analysis

Data entry was carried out using EPI info™. All data were analyzed by SPSS (SPSS Inc, Chicago, IL, USA; Version 15). The averages are reported as Mean ± SD. For all analyses, statistical significance was assessed at a level of 0.05 (2-tailed). In General Linear Model (GLM), menopausal status was inserted as fixed factor and age as covariate to determine the relationship of quantitative variables (as dependent factors) and menopausal status. ANOVA with Tukey Post Hoc test was used to verify exactly in which three levels there were statistically significant differences. In the case of variables without normal distribution shape (like FBG), nonparametric tests were carried out as well but no important differences were shown. Logistic Regression analysis was used to find out whether menopausal status can predict the presence of metabolic syndrome or its components when other effective factors such as age were included in the models.

## Results

Table 1 shows age adjusted relationship of metabolic syndrome risk factors, other biomarkers and medications with menopausal status. Systolic and diastolic blood pressure increased significantly from premenopausal to early menopausal status and continued to increase toward postmenopausal status. Considering total cholesterol, LDL-C and triglyceride, early menopausal and postmenopausal status did not yield significant difference but the values in both were significantly higher in comparison to premenopausal status.

**Table 1:** Age adjusted association of metabolic syndrome and other related biomarkers in menopausal transition (mean ± SD)

Variables	Pre-menopausal (n = 303)	Menopausal (n = 233)	Post-menopausal (n = 987)	P value*
Age (years)	47.2 ± 2.1	51.5 ± 5.8	61.6 ± 8.5	< 0.001
Systolic blood pressure (mmHg)	120.2 ± 20.2	125.2 ± 23	132.4 ± 22.1	0.030
Diastolic blood pressure (mmHg)	77.8 ± 11.8	80.3 ± 12.8	82.5 ± 11.8	0.008
Fasting plasma glucose (mg/dl)	88.7 ± 29.7	91.2 ± 34	95.1 ± 38.2	0.393^§^
2 h-post-prandial glucose (mg/dl)	115 ± 53.4	121 ± 67.4	127.3 ± 70.2	0.011^§^
Total Cholesterol (mg/dl)	209.8 ± 44.6	233.6 ± 54.6	233.3 ± 50.6	< 0.001^†^
LDL-C (mg/dl)	127.2 ± 38.2	142.8 ± 47.3	142.5 ± 43.5	0.004^†^
Triglycerides (mg/dl)	176.1 ± 98.9	213.7 ± 118.1	207.8 ± 104.9	< 0.001^†^
HDL-C (mg/dl)	48.3 ± 10.1	50.4 ± 11	49.5 ± 10.8	0.121
Waist (cm)	97.6 ± 12.5	97.2 ± 13.4	98.5 ± 12.3	0.197
Waist to hip ratio	0.92 ± 0.07	0.93 ± 0.08	0.95 ± 0.07	0.391
Body mass index	28.6 ± 4.7	28.3 ± 5.1	27.6 ± 4.5	0.932
Anti diabetes medication	12(4%)	13(5.6%)	115(11.7%)	0.003
Anti hypertension medication	35(11.6%)	48(20.6%)	324(32.9%)	0.153
Global Dietary Index	0.90 ± 0.23	0.87 ± 0.23	0.84 ± 0.27	0.630
Leisure Time Physical Activity	62.40 ± 82.37	66.19 ± 88.04	62.09 ± 82.54	0.482

* Age adjusted P-value § Adjusted for Anti diabetes medication in addition to age

†Post hoc test did not find significant relationship between early menopausal and Post- Menopausal group

**Figure 1:**
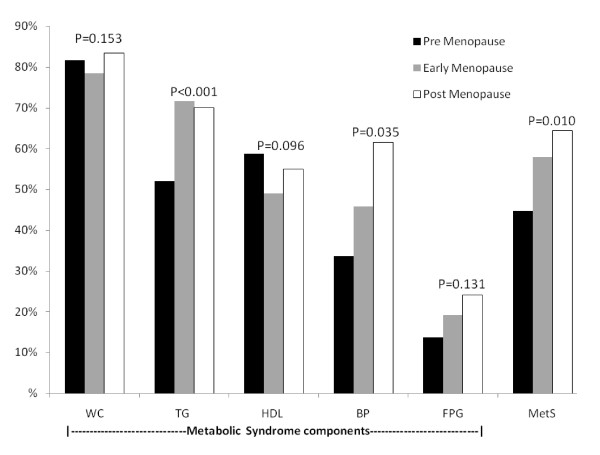
**Age adjusted metabolic syndrome components and metabolic syndrome in menopausal transition (age adjusted P-value for comparison of differences between pre menopause, menopause and post menopause groups)**.

Figure 1 shows the percentage of metabolic syndrome and its components in menopausal transition. Metabolic syndrome was found in 136(44.9%) premenopausal participants and significantly increased to 135(57.9%) and 634(64.3%) in early menopausal and postmenopausal participants respectively (P < 0.001). This association remained significant after adjusting for age (P = 0.010).

Early menopausal status increased risk of metabolic syndrome by 1.64 times

(1.10-2.43, 95%CI) and the odds ratio for postmenopausal status was 1.68 (1.15- 2.45) in comparison to premenopausal status when adjusted for age, Global Dietary Index and Leisure Time Physical Activity. However, only triglyceride and blood pressure components of metabolic syndrome were significantly associated with early menopausal status when the relationships were adjusted for age. Considering Triglyceride component of metabolic syndrome as dependent variable, adjusted Odds Ratio were 2.56(1.68-3.90) and 1.92(1.31-2.83) for early menopausal and postmenopausal status respectively (P < 0.001). On the contrary, for blood pressure the early menopausal status did not contribute significantly to the model when it was adjusted for GDI, leisure time physical activity and age. The model including anti diabetic drug did not change non significant association of FBG and menopausal state. The participants came from both urban and rural areas. The prevalence of metabolic syndrome in urban area was 48.9%, 62.7% and 66% in pre menopausal, early menopausal and postmenopausal groups respectively while these percentages in rural area were 33.7%, 46.4 and 59.5% respectively (P < 0.001 for both groups). Although living in rural area was a protective factor for metabolic syndrome and decreased it 0.67(0.53-9.84) times but adjusted model for area yielded nearly the same association between menopausal status and metabolic syndrome. Moreover, the interaction of menopausal status and area of residence was insignificant (P = 0.296).

## Discussion

As our results show, the prevalence of MetS in the PM women has increased significantly in comparison with menopause and pre-menopause period, even after age adjustment. Moreover, regarding the components of MetS, a significant increase was observed in the frequency of PM women with hypertrigyceridemia and hypertension. However, no significant difference was observed among fasting blood glucose, HDL, and waist circumference (abdominal obesity) with menopausal status when age was included in the model. As all analyses were age adjusted, it means that no further differences other than what was attributable to age were found in these items. The change in metabolic status of the body and the type of fat substitution in different tissues is one of the theories about the incidence of MetS during PM. In pre-menopausal women, fat accumulates in lower extremities to a great extent as a result of estrogen secretion. During menopause the pattern of hormone secretion changes and gradually causes fat accumulation in visceral tissues of abdomen and as a result, central obesity [22]. Many metabolic changes in PM women are related to estrogen secretion decrease and consequent accumulation of abdominal fat. To decrease the risk of MetS and also CVD in PM women, life style modification to control weight, lipid profile, blood pressure and blood glucose is recommended. Besides the abdominal obesity, changes in lipid profile and also insulin resistance are two important factors that are influenced by hormone secretion decrease. However, this is the question: does estrogen have a primary role in MetS incidence or it only masks the genetic effect of MetS incidence and resultant CVD [3,23]. A longitudinal study indicated that weight gain is one of the main changes in PM women. Also, relationship between FSH level and waist circumference increase with the incidence of abdominal obesity has been shown [24]. A nine-year cohort study in various ethnic groups demonstrated that 13.7% of women were affected by MetS in menopause period and all components of MetS changed significantly in the studied women after menopause. This study demonstrated that occurrence of MetS in PM women was the result of testosterone hormone secretion and changes in all components of MetS[25].

In another study, women going pass the menopausal transition experience harmful change in inflammatory mediators and adipokines with visceral obesity [26]. Matthews et al showed increase in lipoproteins at time of menopause [27].

These studies can be reasons for the higher prevalence of MetS by menopause.

Furthermore, Tehran Lipid and Glucose Study (TLGS) showed that the incidence of MetS during PM period as well as all components of MetS in menopausal transition period increase [28].

Nevertheless, results of our study demonstrate that although there was not significant difference in abdominal obesity and most components of MetS in the studies women, the risk of MetS increased in menopausal transition period. This finding is different from the results of most studies which indicate that metabolic changes and consequent lipid accumulation and blood glucose changes during PM are responsible for MetS. In spite of similarities between the incidence of MetS incidence in menopausal transition period in TLGS and this study, the changes of mean value of metabolic syndrome components in two studies did not follow the same pattern. TLGS did not study the components of MetS as qualitative variables. In addition, the sampled population in this study came from a greater area which covered both urban and rural population, while in TLGS sampling was done only in a part of Tehran. Prevalence of MetS in this study was higher than seen in western countries in both sexes (20.9% of men had MetS that not shown). The etiology of it explained in Sarrafzadegan et al study [29]. One of the advantages of this study is studying life style of the three studies groups of women to investigate the effect of this factor on incidence of MetS and its components. Some previous studies did not include this aspect. The three groups were not different, considering physical exercise and nutrition index in menopausal transition independent of age.

Moreover, adding the two main factors of life style and age to the model of estimating the risk of MetS did not change the risk in menopausal and postmenopausal period. This is indicative of independence MetS of age and life style.

In addition, of the components of MetS, only hypertriglyceridemia was significantly in accordance with this model. However, among the quantitative variable without being adjusted for nutrition and physical exercise, only mean blood pressure and triglyceride level changes reached the significant level.

Absence of significant difference in incidence of MetS components except for one of them and the significant difference of incidence of MetS in the three groups indicate that MetS is something more than the simple clustering of the factors.

This is one of the few studies which show no difference in obesity and fasting blood glucose independent of age between premenopausal and post menopause.

This finding suggests that besides the aforementioned factors, other effective factors such as genetic or environmental ones contribute to the pathogenesis of the disease, which shows their effect in various ethnic groups differently. As it was suggested in the study fulfilled by Carr, it can be mentioned that the presence of estrogen have a genetic masking effect, rather than a primary role in MetS control. Thus, the role will be removed by elimination of estrogen and the incidence of MetS will be increased, in spite of absence of central obesity [3].

Considering some differences between the findings of current study with results of other studies and shortage of similar studies in Middle East, carrying out longitudinal studies on women of different ethnic groups in different regions and study of MetS and its components in menopausal transition in addition to genetic, hormonal, climactic, and environmental factors can lead to a better understanding of the above mentioned phenomena.

## Competing interests

The authors declare that they have no competing interests.

## Authors' contributions

RH, MS, NS participated in the design of the study. MS, MT, KR performed the statistical analysis. RH, KR, NM participated in data collection. All authors read and approved the final manuscript.
